# Predicting recurrence of depression using lifelog data: an explanatory feasibility study with a panel VAR approach

**DOI:** 10.1186/s12888-019-2382-2

**Published:** 2019-12-11

**Authors:** Narimasa Kumagai, Aran Tajika, Akio Hasegawa, Nao Kawanishi, Masaru Horikoshi, Shinji Shimodera, Ken’ichi Kurata, Bun Chino, Toshi A. Furukawa

**Affiliations:** 1 Department of Economics, Seinan Gakuin University, 6-2-92, Nishijin, Sawara-ku, Fukuoka, 814-8511 Japan; 2 Department of Psychiatry, Kyoto University Hospital, 54 Shogoin-Kawahara-cho, Sakyo-ku, Kyoto, 606-8507 Japan; 3 Advanced Telecommunications Research Institute International, 2-2-2 Hikaridai, Seika-cho, Soraku-gun, Kyoto, 619-0288 Japan; 4 Sonas Inc., 6F, Grace Imas Building, 5-24-2, Hongo, Bunkyo-ku, Tokyo, 113-0033 Japan; 5 National Center for Cognitive Behavior Therapy and Research, National Center of Neurology and Psychiatry, 4-1-1 Ogawahigashi-cho, Kodaira, Tokyo, 187-8553 Japan; 6 Ginza Shimodera Clinic, 8B-6-9-6 Ginza Chuo Ward, Tokyo, 104-0061 Japan; 7 Department of Health Promotion and Human Behavior, Kyoto University Graduate School of Medicine / School of Public Health, Yoshida Konoe-cho, Sakyo-ku, Kyoto, 606-8501 Japan; 8 Kabe Mental Health Clinic, 4-6-2 Kabe, Asakita-ku, Hiroshima, 731-0221 Japan; 9 Ginza Taimei Clinic, 5-1-15 Ginza, Chuou-ku, Tokyo, 104-0061 Japan

**Keywords:** Depression, Kessler psychological distress scale, Kurashi-app, Lifelog, Long sleep time, Panel vector autoregressive model, Patient health Questionnaire-9

## Abstract

**Background:**

Although depression has a high rate of recurrence, no prior studies have established a method that could identify the warning signs of its recurrence.

**Methods:**

We collected digital data consisting of individual activity records such as location or mobility information (lifelog data) from 89 patients who were on maintenance therapy for depression for a year, using a smartphone application and a wearable device. We assessed depression and its recurrence using both the Kessler Psychological Distress Scale (K6) and the Patient Health Questionnaire-9.

**Results:**

A panel vector autoregressive analysis indicated that long sleep time was a important risk factor for the recurrence of depression. Long sleep predicted the recurrence of depression after 3 weeks.

**Conclusions:**

The panel vector autoregressive approach can identify the warning signs of depression recurrence; however, the convenient sampling of the present cohort may limit the scope towards drawing a generalised conclusion.

## Background

Depression has a high rate of recurrence. Epidemiological and clinical evidence suggests that major depressive disorder typically follows a recurrent course, with a third to half of the patients relapsing within 1 year of discontinuation of treatment [1]. The greater the number of prior depressive episodes, the higher is the probability of a future recurrence [2, 3]. Therefore, it is very important to identify the warning signs of recurrence early in order to prevent it.

A large number of studies have investigated the environmental factors that predict depression recurrence. Social support including marriage may reduce the recurrence risk [4], and women are more vulnerable to depression recurrence in midlife [5, 6]. However, factors such as educational attainment, socioeconomic status, life events, and number of children have shown no significant association with depression recurrence [6]. Key lifestyle factors that may predict recurrence are still poorly understood. One study involving healthy individuals found that a more irregular social rhythm—not going to bed or eating meals at a similar time every day—was predictive of dismal mental health [7]. In recent years, it has become easier to obtain lifelog data from smartphones and wearable devices. Lifelog refers to digital data of individual activity records such as location information or mobility information. Nevertheless, no study has investigated the relationship between depression recurrence and the daily activities of patients in remission.

Statistical analysis is often laden with ambiguities when investigating the relationship between mental health status and lifestyle factors (e.g., sleeping habits). Cox proportional-hazards models are commonly used when examining time to recurrence [2, 4, 8–10] or when testing for moderation of maintenance-treatment effects on recurrence [11]. However, survival analysis using Cox proportional-hazards models cannot take into account the bidirectional relationships between lifestyle factors and poor mental health status. For example, insomnia can increase depression risk and vice versa [12]. On one hand, the value of weekly Kessler Psychological Distress Scale (K6) in the current period is likely to correlate with its value in the previous period; whereas on the other hand, bidirectional causal relationships between weekly K6 and its closely related variables might be discovered. The changes in lifestyle can be seen as the predictors of future recurrence of depression, or as precursors that are early signs of depression recurrence. The estimation function should include two aspects of predictors and precursors, regardless of whether true roles are independent predictors or not.

Therefore, multivariate regression such as vector autoregressive (VAR) models must be used when analysing possible risk factors of depression recurrence. The VAR models fit each dependent variable on past lags of itself.

In the present study, we examined whether the change of activity record on weekly basis calculated from lifelog data collected via smartphone and a wearable device, such as time spent sleeping, exposure to ultraviolet (UV) light, number of meals, etc., could predict the recurrence of depression among patients with major depression remission. We estimated panel VAR (PVAR) models considering both directions of the relationship between risk factors of recurrence and poor mental health status.

This manuscript is organised as follows. Section 2 outlines the characteristics of the data collected through lifelogging applications via a smartphone app and a wearable device. In this section, the variables of interest and the empirical methods are described. Section 3 reports the estimation results of the PVAR models. Section 4 discusses the specificity of this study. Finally, Section 5 contains the conclusions.

## Methods

This study explored the dynamic interdependencies between poor mental health status and lifestyle factors. We used PVAR models that have lags of all endogenous variables and analysed the weekly interdependencies among variables of interest.

### Procedures

We used a smartphone (iPhone, Apple inc.) app called Kurashi-app (“kurashi” means “life” in Japanese) and a wearable device (Silmee W20, TDK Co. Ltd., Japan) to collect lifelog data from 89 patients who had suffered from major depression, but were then in remission. We collected the activity diaries of the patients over a period of 1 year.

There are some advantages of having patients record their activity diaries via a smartphone. First, it is easier for patients to record their activity, regardless of time and place, compared to the conventional method of pen and paper. Secondly, utilizing the lifelogs, patients can record their activity diaries more easily. The Kurashi-app collects individual’s lifelogs via smartphone and estimates their activities based on them. It predicts 16 types of activities from lifelog data of location information, mobility information, and steps information and displays them on screen. The patients are expected to check them every day, and when the prediction is incorrect, they can rectify it. Consequently, the precision of the prediction improves. The recordings of the 16 activities on Kurashi-app can therefore be regarded as semi-automated self-reports. The system is expected to increase precision and to reduce burden on the part of the participants. When being presented with their estimated activities, users can be helped to record activity diaries even when they cannot distinctly remember certain activities from their daily lives [13].

The Kurashi-app includes 16 kinds of activities: meeting friends or family, bath/shower, childcare/caregiving, commuting, domestic work, exercise, hospital, meal, shopping, sitting idly, sleep, study/work, hobby/entertainment/learning, TV/DVD/game/music, reading/newspaper/magazine, and other activities. These 16 activities were selected from the classification of the Basic Survey on Social Life (Shakai seikatsu kihon chosa) by the Statistics Bureau of the Ministry of Internal Affairs and Communications in Japan. Sitting idly is included because “time spending vaguely without doing anything in particular” is considered important with regard to poor mental health status. The Kurashi-app extracts clusters of activities where patients stay for more than 30 min at a time.

Simee W20 is a wrist watch type wearable device which can collect UV data automatically, in addition to location and mobility information.

We collected the activity diaries of each patient for 1 year. Outpatients were recruited at four university hospitals and their associated hospitals and clinics. Kyoto University was the central secretariat, and the 4 university hospitals were Kochi University, Hiroshima University, Nagoya City University, and Toho University. We recruited a hundred patients in total into the study between October 2016 and March 2017. Ten patients withdrew themselves from the study; while one patient did not meet the inclusion criteria and was therefore excluded. Inclusion criteria were as follows: (1) age between 22 and 69 years; (2) meet DSM-5 criteria for major depressive disorder, recurrent; (3) in remission as defined by the Beck Depression Inventory-II score of 9 or less [14]; (4) with or without anxiety disorder or dysthymia; (5) able to use a mobile phone; (6) able and willing to participate in the study. We excluded patients with bipolar disorder, substance use disorder, psychosis, and personality disorder.

We used two screening and diagnostic tools to assess depression, K6 and the Patient Health Questionnaire-9 (PHQ-9). Using the Kurashi-app, patients completed the K6 by themselves once a week. However, such recordings are prone to lapses because they rely on self-reports on the smartphone. At the doctor’s consultation every 4 weeks, clinical study coordinators of Kyoto University assessed the patients through the PHQ-9 on the telephone. When the patients failed to visit the doctor, we contacted them by telephone to make the monthly assessments with PHQ-9 in order to minimise carelessness.

The K6 is a six-item screening instrument assessing psychological distress developed by Kessler and his colleagues [15]. Respondents rated how frequently they had experienced the following six symptoms over the past 7 days: a) feeling nervous, b) feeling hopeless, c) feeling restless or fidgety, d) feeling depressed to the point that nothing could cheer you up, e) feeling everything was an effort, and f) feeling worthless. Respondents rated each item using a 5-point scale: 0 (“none of the time”), 1 (“a little of the time”), 2 (“some of the time”), 3 (“most of the time”), or 4 (“all of the time”). Responses to the six items were summed to yield a K6 score between 0 and 24, with higher scores indicating a greater tendency towards mental illness. Using the receiver operating characteristic curve, Prochaska et al. [16] identified a K6 score ≥ 5 as the optimal cut-off point indicative of moderate mental distress. The coefficient of correlation between K6 and the Hamilton Depression Rating Scale was 0.516 at the 1% significant level [17].

The PHQ-9 questionnaire asks respondents how frequently they have experienced the following nine symptoms over the past 2 weeks: a) having little interest or pleasure in doing things, b) feeling depressed or hopeless, c) having trouble staying asleep or sleeping too much, d) feeling tired, e) having poor appetite or overeating, f) feeling bad about oneself, g) having trouble concentrating on things, h) moving or speaking so slowly that other people could have noticed, i) having thoughts that you would be better off dead. Respondents rated each item using a 4-point scale: 0 (“not at all”), 1 (“several days”), 2 (“more than half the days”), or 3 (“nearly every day”). The PHQ-9 is commonly used to screen for depression with 10 as the cut-off score; a score of 10–14 indicates moderate depression, 15–19 moderately severe depression, and 20–27 severe depression. We have slightly modified the time frame for PHQ-9 in this study and asked the participants to rate their symptoms during the worst two weeks of the past month, in order to increase sensitivity to detect a depressed episode between the monthly assessments. The item responses on the PHQ-9 exhibited the same mathematical pattern as the other depression screening scales such as K6 and the Center for Epidemiological Studies Depression Scale [18].

Daily chart of the 16 activities were visualised on the Kurashi-app, and all participants could check the data themselves at any time. The data from wearable device could be checked by connecting the device to their iPhone (this task was voluntary).

Since participants experienced some recurrence of depression, their motivations to know the sign of recurrence was high. We paid 5000 JPY (= about 47 USD) per month to the participants for 1 year. About half of the participants had their own iPhone and downloaded the app. To the remaining half, we lent our study iPhones, which were returned after the follow up period. We also lent wearable devices to all participants. The patients were expected to don the wearable device except when they bathed. We also accepted it when some patients preferred not to wear it while asleep. All data obtained from the app and wearable device were uploaded to the database server at Kyoto University. We monitored the adherence of the participants and reminded them when the adherence dropped during their monthly visits to the clinics/hospitals.

### Collected data

We analysed the data of K6 score in order to observe weekly change of mood. Because K6 is a self-reported questionnaire on the smartphone, some participants forgot to enter their responses on the Kurashi-app weekly. When K6 data is missing, the lagged variables of the PVAR models do not show real-time differences. In order to avoid this problem, the researchers must supplement missing data. Assuming that data is missing at random (MAR), to supplement missing K6 data, we used the PHQ-9 score as an explanatory variable of the multiple regression equation. Daily missing data was not associated with the recurrence of depression, and we used its imputed series for weekly data series.

When dealing with MAR data, we can consider that the probability distribution of missing data is independent of that of non-observational data. We conducted Little’s CDM (covariate-dependent missingness) test [19] as a special case of MAR. The CDM test gave a *p*-value 0.102 and the hypothesis that the variables of interest are MCAR (missing completely at random) were not rejected at the 5% significance level. Therefore, the estimate is biased when one ignores the missing data [20]. We corrected this bias by estimating the regression equation using auxiliary variables. A previous study showed that the use of many auxiliary variables may contribute to satisfy the premise of MAR [21].

The regression equation approach may underestimate the standard deviation of the true value. However, Stata (ver. 15) cannot run PVAR models after multiple imputations. We thus estimated the multiple regression equation and imputed missing variables. The long length of activity diaries may increase the number of recurrence episodes over the sample period. Considering this, researchers must pay attention to heteroskedasticity problems. We used the number of weeks from entry in the study as the analytic weight of the regression equation. This variable is inversely proportional to the variance of observations. Lifelog data were aggregated by the week. Explanatory variables of the regression equation to impute K6 data were as follows: PHQ-9 score, mean of sleep hours during the past week, long sleep time, short sleep time, gender, educational attainment, occupational status, and marital status.

We selected the variables of the PVAR models as follows. The first candidate variables were enough time spent sleeping and exposure to UV light. These are good lifestyle factors recommended by Sarris et al. [12]. The second candidate variables were selected using a two-sample t-test for difference of means. The homogeneity of variance was assumed. The two-sample t-test (the sample was divided between those with long sleep time/short sleep time and without) showed differences for five variables: meal, sitting idly, study/work, domestic work, and exercise. Finally, we calculated the correlation coefficient between K6 scores and these five activity variables, and selected two as explanatory variables of the PVAR models. The correlation coefficients of the two variables with K6 scores were 0.229 for sitting idly and 0.199 for the number of times lunch was not eaten.

The UV light exposure data were collected every minute by a wearable device Silmee W20. We defined a missing UV light value when collected data was below 80% of 1440 min (1152 min). Major reasons for missing out on UV light data were depleting battery life or restrained donning of the wearing device because of periodic irritation. Considering the differences in eating habits, we calculated a standardised variable of the number of times lunch was not eaten and used it as an explanatory variable of the PVAR models. We used standardised variables in order to accommodate patient heterogeneity that may account for large portion of total variances of key variables when using non-standardised variables. Since the standard deviation of the number of times lunch was not eaten was a large value of 2.46, we considered that the raw variable did not capture the differences in eating habits. Like the procedure for K6 scores, we imputed missing values of UV light and the standardised variable of the number of times lunch was not eaten.

### PVAR model

As a key dependent variable of weekly K6, we considered a 5-variate PVAR of order p with panel-specific fixed effects represented by the following system of linear equations:

1 $$ {Y}_{it=}{Y}_{it-1}{A}_1+{Y}_{it-2}{A}_2+\cdots +{Y}_{it-p}{A}_p+{X}_{it}B+{v}_{it}+{e}_{it} $$

where *Y*_*it*_ is a (1 × 5) vector of dependent variables; *X*_*it*_ is a (1 × q) vector of exogenous covariates; *v*_*it*_ and *e*_*it*_ are (1 × 5) vectors of dependent variable-specific fixed-effects and idiosyncratic errors, respectively. The (5 × 5) matrices *A*_*1*_, *A*_*2*_,…, *A*_*p*_ and the (q × 5) matrix *B* are parameters to be estimated.

With the presence of lagged dependent variables in the right-hand side of the system of equations, estimates would be biased even with a large *N* [22]. Fixed-effects estimation tends to underrate the predictions of the coefficient of the lagged dependent variables. Taking these problems into consideration, following the procedure of Michael-Abrigo and Love [23], we used unbalanced panel data and estimated PVAR models by fitting a multivariate panel regression of each dependent variable on lags of itself and on exogenous variables. The estimation was done using the generalised method of moments (GMM). Because the presence of a unit root will invalidate the GMM specification, the estimates of the PVAR model must satisfy the stability condition. If all the eigenvalues lie inside the unit circle, the stability condition of the PVAR model is satisfied and the PVAR model is invertible.

We specified the PVAR model as follows. First, using the overall coefficient of determination (CD), we specified the maximum lag order to be included in the PVAR model. As explained below in section 3.1, the PVAR model consisted of five dependent variables as follows: the natural logarithm of {(K6 + 1)/square root of the number of episodes}, dummy variable of long sleep time or short sleep time, standardised variable of the number of times lunch was not eaten, natural logarithm of standardised variable of UV light, and dummy variable of sitting idly. Because the K6 repeated with relatively high frequency may cause respondent’s “learning curve” reaction, we used the square root of the number of previous episodes to control for each patient’s past experiences of depression. All the patients suffered from recurrent depression and those with increased numbers of previous episodes tended to report, on average, higher K6 scores. In order to balance the sensitivity of K6 scores across the subjects with variable number of previous episodes, we divided their natural logarithm of (K6 + 1) scores by the square root of their number of previous episodes.

Second, we confirmed the stability condition of the estimated PVAR model. Third, based on the value of CD shown in Table 1, we decided that the maximum lag order was 4. The CD captures the proportion of variation explained by the PVAR model as follows:

**Table 1:** Maximum lag order of PVAR

Number of participants = 87	
Average number of weeks = 41.95	
Lag	CD
1	0.98562
2	0.98827
3	0.98933
4	0.98972
5	0.98762
6	0.96484

*CD* coefficient of determination, *T* Observations per panel

CD = 1–(determinant of covariance matrix of idiosyncratic errors/determinant of unconstrained covariance matrix of the dependent variables).

## Results

### Patient characteristics and PVAR variables

Initially we recruited 100 patients, but one patient was found not to have met the inclusion criteria at baseline and was therefore excluded. Of the 99 remaining patients, 10 dropped out. The reasons were as follows: too burdensome (*n* = 6), house relocation (*n* = 2), recovered (n = 1), and lost to follow up (*n* = 1). While 92 % of activity diaries during the sample period were recorded, the patients corrected one third of estimated activities by the Kurashi-app. Table 2 shows the characteristics of patients at the baseline and the variables of the PVAR models are shown in Table 3. The mean age of patients at the time of entry was 44.3 years; 74% of patients had received education after high school graduation. The largest proportion had regular employment (54%), followed by inactive persons (16%) and part-time workers (13%). Married persons accounted for 54% of the sample, followed by those who never married (33%) and divorced persons (12%). The mean age at the first depression episode was 34.9 years. The mean time period from the first episode was 9.4 years. The mean number of depression episodes was 2.5. The correlation between the time from the first depression episode and the number of episodes was significant at the 1% level. The correlation coefficient was 0.38 for women and 0.33 for men.

**Table 2:** Characteristics of patients

Variables	Number (%) or Mean (SD)
Gender (Male)	49 (55.1)
Age	44.3 (10.8)
*Educational background*	
Junior high school	3 (3.4)
High school	20 (22.5)
University, etc.	66 (74.2)
*Occupational status*	
Regular worker	48 (53.9)
Part-time worker	12 (13.5)
Leave	3 (3.4)
Homemaker	6 (6.7)
Retired	3 (3.4)
Inactive	14 (15.7)
Other	3 (3.4)
*Marital status*	
Never married	29 (32.6)
Divorced	11 (12.4)
Widowed	1 (1.1)
Married	48 (53.9)
*First depression episode*	
Age at first episode	34.9 (11.6)
Years from first episode	9.4 (5.8)
Number of episodes	2.5 (1.7)

*N* = 89

**Table 3:** Dependent variables of panel vector autoregressive (PVAR) models

Variables	*N*	Mean	SD	Min	Max
K6	4830	4.19	4.35	0.0	24.0
Natural logarithm of (K6 + 1)	4830	1.30	0.87	0.0	3.22
Daily sleep hours during the past week					
Mean	4476	7.99	1.61	0.9	20.1
SD	4401	1.48	1.05	0	8.5
Coefficient of variation	4401	0.19	0.14	0	2.6
Dummy variable for long sleep	4863	0.04	0.19	0	1
Dummy variable for short sleep	4863	0.04	0.21	0	1
Daily hours sitting idly during the past week	4501	1.13	1.43	0	14.5
Daily UV light during the past week	4840	16.47	16.66	0	136.6
Number of days of not eating lunch	4863	2.48	2.46	0	7

It is well known that there is a positive relationship between sleep disorders and depression [24]. Taking this relationship into account, we defined dummy variables of both long sleep time and short sleep time. Hours of sleep were considered the total hours slept from noon of the previous day to noon of the current day. The dummy variable of long sleep time took the value of 1 when the number of hours of sleep was higher than the sum of mean hours of sleep over the past 7 days and its standard deviation. In contrast, the dummy variable of short sleep time took the value of 1 when the number of hours of sleep was lower than the difference between the mean hours of sleep over the past 7 days and its standard deviation. Long sleep and short sleep were defined according to the sleep time which participants had self-reported on Kurashi-app.

### Estimation results

**Fig. 1:**
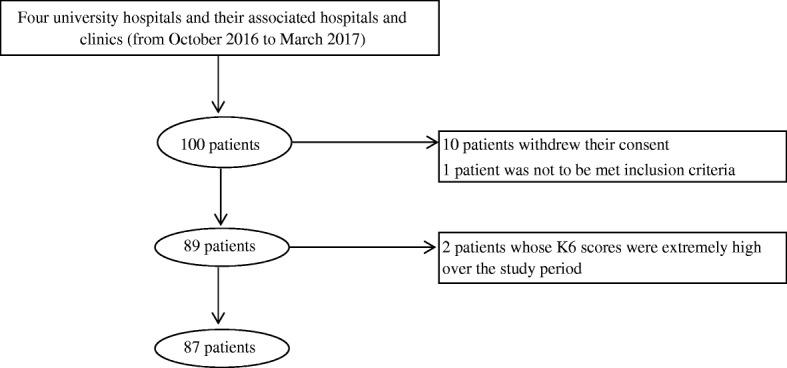
Flow chart of recruited patients. Note: Inclusion criteria are as follows: (1) Age between 22 and 69 years; meet DSM-5 criteria for major depressive disorder recurrent episode; (3) Beck Depression Inventory-II score 0–9 (which means remission); (4) with or without anxiety disorder or dysthymia; (5) able to use mobile phone; (6) able and willing to participate in the study

We excluded two patients whose K6 scores were extremely high through the study period, and thus used 87 samples (Average Observations per panel = 44.33 weeks) for the PVAR model estimation (Fig. 1). Because invariant variables such as educational attainment are excluded when estimating PVAR models, we used a seasonal dummy variable and a pseudo-positive dummy variable as exogenous variables. The seasonal dummy variable took the value of 1 if the month during the study period was December or January, and 0 otherwise. The pseudo-positive dummy variable took the value of 1 if K6 score > 9 and PHQ-9 score < 5. It means a false alarm of recurrence of depression. The prevalence of pseudo-positive was 0.3% (17/4863).

The PVAR model using the whole sample, which included five dependent variables and four lags, satisfied the stability condition. The estimated coefficient indicated that 3-week lagged long sleep increased the K6 score in the present week. Results of Model I (*N* = 87) indicated that long sleep time in patients predicted the recurrence of depression after 3 weeks (Table 4). The estimated coefficient of this week-lagged long sleep was 0.172. This implies that long sleep time increased the K6 score from 5 to 6.126 after 3 weeks. Model I had positive lagged effects of long sleep on K6 and not eating lunch, and negative lagged effects of K6 on not eating lunch (not shown in Table 4).

**Table 4:** PVAR model I (*N* = 3857)

Selected variables	Natural log of {(K6 + 1)/square root of the number of episodes}	Number of times not eating lunch (standardized)	Natural log of UV light	Long sleep	Sitting idly
Not eating lunch (−1)	0.0117	0.338***	0.00208	−0.0385	0.0864
	(0.0431)	(0.0430)	(0.0116)	(0.0465)	(0.0630)
Not eating lunch (−2)	−0.00159	0.191***	−0.00408	− 0.0415	0.0740
	(0.0364)	(0.0371)	(0.0103)	(0.0402)	(0.0571)
Not eating lunch (−3)	−0.0581*	0.111***	0.00909	−0.0301	0.0132
	(0.0348)	(0.0353)	(0.00885)	(0.0355)	(0.0509)
Not eating lunch (−4)	0.00318	0.0810***	−0.0106	−0.00718	0.0219
	(0.0304)	(0.0308)	(0.00793)	(0.0336)	(0.0441)
UV (−1)	− 0.0411**	−0.0628***	0.00183	0.450***	0.0495**
	(0.0185)	(0.0192)	(0.00523)	(0.0248)	(0.0251)
UV (−2)	0.00857	−0.0205	0.00640	0.164***	0.0498**
	(0.0166)	(0.0170)	(0.00521)	(0.0238)	(0.0235)
UV (−3)	0.00510	−0.0344**	− 0.00297	0.109***	− 0.00320
	(0.0155)	(0.0162)	(0.00492)	(0.0223)	(0.0221)
UV (−4)	−0.0229	− 0.0358**	− 0.00667	0.135***	0.0333
	(0.0150)	(0.0157)	(0.00512)	(0.0218)	(0.0205)
Long sleep (−1)	0.0658	0.173*	0.280***	0.118	−0.144
	(0.0895)	(0.0899)	(0.0397)	(0.103)	(0.105)
Long sleep (−2)	0.0807	0.287***	0.123***	0.0366	0.0359
	(0.0757)	(0.0903)	(0.0345)	(0.0959)	(0.115)
Long sleep (−3)	0.172**	0.228**	0.142***	−0.0897	−0.0899
	(0.0829)	(0.0901)	(0.0359)	(0.0884)	(0.0909)
Long sleep (−4)	0.0933	0.209**	0.109***	−0.0217	0.0441
	(0.0830)	(0.0841)	(0.0343)	(0.0900)	(0.107)
Sitting idly (−1)	−0.00942	− 0.0551**	− 0.000905	0.0327	0.327***
	(0.0196)	(0.0215)	(0.00506)	(0.0235)	(0.0518)
Sitting idly (−2)	−0.00372	−0.0298*	0.00194	0.0235	0.120***
	(0.0161)	(0.0180)	(0.00466)	(0.0203)	(0.0376)
Sitting idly (−3)	0.00581	−0.0308*	0.000352	0.0320*	0.0962***
	(0.0151)	(0.0162)	(0.00475)	(0.0186)	(0.0366)
Sitting idly (−4)	−0.00756	−0.0343**	0.000806	−0.0228	0.0911**
	(0.0137)	(0.0168)	(0.00443)	(0.0194)	(0.0404)
Seasonal dummy variable	−0.0102	−0.0802*	0.0144	−0.252***	0.0773
	(0.0402)	(0.0416)	(0.0109)	(0.0488)	(0.0575)
Pseudo-positive dummy variable	1.984***	0.590	0.108	−0.493**	0.283
	(0.197)	(0.596)	(0.145)	(0.233)	(0.200)

Number of participants = 87

Average number of weeks = 44.33

Lagged dependent variables are included

Standard errors in parentheses. *** *p* < 0.01, ** *p* < 0.05, * *p* < 0.1

The prevalence of long sleep time was about 6% in the patients aged 50–59 years, while it was almost 3% in the other age groups. Patients aged 50–59 years had a higher regular employee ratio (72.2%), and the proportion of regular employees in the other age groups was 50.3%. Because there was an intergenerational difference in both the prevalence of long sleep time and regular employee ratio, we used sub-samples. We estimated two PVAR models: (II) sample excluding patients aged 50–59 years (*N* = 69) and (III) patients aged 50–59 years (*N* = 18).

Model II with a pseudo-positive dummy variable consisted of five dependent variables, which satisfied the stability condition (lags = 4, CD = 0.987953). On the other hand, model III without a pseudo-positive dummy variable consisted of four dependent variables, which satisfied the stability condition (lags = 4, CD = 0.994561). A pseudo-positive dummy variable was not significant in model III.

Tables 5 and 6 show the estimation results of models II and III. We found that long sleep time was an important risk factor for the recurrence of depression. Model II excluding patients aged 50–59 years suggested two aspects of long sleep time as a strong predictor of depression recurrence. First, we found a replicated effect including positive lagged effects of long sleep on K6 and not eating lunch, and negative lagged effects of K6 on long sleep and not eating lunch. Secondly, long sleep time was a superior predictor of depression recurrence, compared to other factors because not eating lunch did not have a significant effect on K6 at the 5% level.

**Table 5:** PVAR model II (*N* = 3023)

Selected variables	Natural log of {(K6 + 1)/square root of the number of episodes}	Number of times not eating lunch (standardized)	Natural log of UV light	Long sleep	Sitting idly
Not eating lunch (−1)	0.00237	0.295***	−0.0364	− 0.00678	0.0985
	(0.0462)	(0.0459)	(0.0515)	(0.0124)	(0.0673)
Not eating lunch (−2)	−0.0225	0.167***	−0.00376	− 0.0165	0.0553
	(0.0393)	(0.0392)	(0.0435)	(0.0107)	(0.0608)
Not eating lunch (−3)	−0.0734*	0.0875**	−0.0256	−0.000261	0.00743
	(0.0378)	(0.0374)	(0.0384)	(0.00932)	(0.0549)
Not eating lunch (−4)	− 0.00421	0.0561*	−0.0200	− 0.0101	0.0209
	(0.0328)	(0.0325)	(0.0359)	(0.00853)	(0.0458)
UV (−1)	−0.0394*	−0.0726***	0.421***	0.00492	0.0631**
	(0.0224)	(0.0238)	(0.0295)	(0.00599)	(0.0289)
UV (−2)	0.00750	−0.0187	0.161***	0.00253	0.0285
	(0.0200)	(0.0203)	(0.0271)	(0.00584)	(0.0268)
UV (−3)	0.00711	−0.0440**	0.116***	−0.00524	− 0.0106
	(0.0188)	(0.0196)	(0.0257)	(0.00552)	(0.0252)
UV (−4)	−0.0181	−0.0490**	0.142***	−0.00550	0.0370
	(0.0185)	(0.0193)	(0.0249)	(0.00557)	(0.0233)
Long sleep (−1)	0.126	0.214*	0.152	0.239***	−0.111
	(0.113)	(0.115)	(0.115)	(0.0461)	(0.134)
Long sleep (−2)	0.0804	0.266***	0.0850	0.0823**	0.0514
	(0.0904)	(0.102)	(0.0958)	(0.0376)	(0.120)
Long sleep (−3)	0.271***	0.351***	−0.111	0.128***	−0.0567
	(0.0999)	(0.107)	(0.102)	(0.0417)	(0.113)
Long sleep (−4)	0.104	0.282***	0.0973	0.0614*	0.0433
	(0.103)	(0.0981)	(0.0964)	(0.0365)	(0.133)
Sitting idly (− 1)	−0.0134	− 0.0508**	0.0346	−0.00207	0.357***
	(0.0211)	(0.0231)	(0.0238)	(0.00530)	(0.0611)
Sitting idly (− 2)	− 0.0115	− 0.0332*	0.0302	0.00375	0.104**
	(0.0171)	(0.0188)	(0.0207)	(0.00513)	(0.0446)
Sitting idly (−3)	0.00236	−0.0250	0.0306	−0.00170	0.114***
	(0.0159)	(0.0167)	(0.0195)	(0.00441)	(0.0436)
Sitting idly (−4)	− 0.00217	− 0.0399**	− 0.0174	−0.00554	0.0935*
	(0.0145)	(0.0176)	(0.0190)	(0.00438)	(0.0493)
Seasonal dummy variable	0.00857	−0.111**	−0.246***	0.0175	0.0604
	(0.0491)	(0.0513)	(0.0560)	(0.0120)	(0.0649)
Pseudo-positive dummy variable	1.969***	0.737	−0.517*	0.101	−0.0780
	(0.227)	(0.721)	(0.311)	(0.154)	(0.230)

Number of participants = 69

Average number of weeks = 43.81

Lagged dependent variables are included

Standard errors in parentheses. *** *p* < 0.01, ** *p* < 0.05, * *p* < 0.1

**Table 6:** PVAR model III (*N* = 834)

Selected variables	Natural log of {(K6 + 1)/square root of the number of episodes}	Number of times not eating lunch (standardized)	Natural log of UV light	Long sleep	Sitting idly
Not eating lunch (−1)	− 0.0535	0.302***	−0.00145	− 0.000906	− 0.0169
	(0.0709)	(0.0940)	(0.0676)	(0.0167)	(0.0930)
Not eating lunch (−2)	0.00318	0.152*	−0.172**	0.0294	0.140
	(0.0602)	(0.0913)	(0.0732)	(0.0231)	(0.0913)
Not eating lunch (−3)	−0.0583	0.0996	−0.0358	0.0304*	0.0537
	(0.0635)	(0.0912)	(0.0680)	(0.0180)	(0.0823)
Not eating lunch (−4)	−0.152	0.0673	−0.0545	− 0.0457**	−0.0129
	(0.135)	(0.0816)	(0.107)	(0.0216)	(0.162)
UV (−1)	−0.0632	−0.0265	0.523***	−0.00834	− 0.0544
	(0.0441)	(0.0376)	(0.0578)	(0.0113)	(0.0574)
UV (−2)	−0.00312	−0.0180	0.127**	0.0195	0.125**
	(0.0391)	(0.0333)	(0.0599)	(0.0119)	(0.0571)
UV (−3)	−0.000449	− 0.000159	0.0436	− 0.00159	0.0194
	(0.0279)	(0.0330)	(0.0432)	(0.0111)	(0.0517)
UV (−4)	−0.0529**	−0.00642	0.130***	−0.0102	0.00285
	(0.0240)	(0.0299)	(0.0481)	(0.0107)	(0.0476)
Long sleep (−1)	−0.0295	0.0491	0.0586	0.344***	−0.183
	(0.108)	(0.124)	(0.173)	(0.0732)	(0.157)
Long sleep (−2)	0.204	0.211	−0.0162	0.208***	0.0810
	(0.128)	(0.130)	(0.181)	(0.0687)	(0.220)
Long sleep (−3)	−0.0191	−0.0858	0.127	0.117*	−0.173
	(0.130)	(0.141)	(0.140)	(0.0698)	(0.133)
Long sleep (−4)	0.191*	−0.0327	−0.218	0.203***	0.0697
	(0.105)	(0.126)	(0.175)	(0.0756)	(0.149)
Sitting idly (−1)	0.0317	−0.0107	0.0145	0.0121	0.165***
	(0.0350)	(0.0409)	(0.0543)	(0.0140)	(0.0603)
Sitting idly (−2)	−0.00850	0.0431	−0.0792	− 0.0134	0.159
	(0.0862)	(0.0464)	(0.0816)	(0.0167)	(0.110)
Sitting idly (−3)	−0.00393	0.00114	−0.00544	0.00122	0.000576
	(0.0516)	(0.0399)	(0.0533)	(0.0133)	(0.0645)
Sitting idly (−4)	−0.00854	0.0300	−0.0495	0.0234*	0.0202
	(0.0285)	(0.0395)	(0.0455)	(0.0130)	(0.0535)
Seasonal dummy variable	−0.126**	0.0424	−0.270***	− 0.00152	0.142
	(0.0633)	(0.0875)	(0.0992)	(0.0157)	(0.107)
Pseudo-positive dummy variable	12.66	−3.844	15.84	3.057	−5.221
	(21.12)	(14.26)	(13.99)	(2.974)	(22.97)

Number of participants = 18

Average number of weeks = 46.33

Lagged dependent variables are included

Standard errors in parentheses. *** *p* < 0.01, ** *p* < 0.05, * *p* < 0.1

Results of PVAR indicate that a long sleep in patients aged 50–59 years predicted the recurrence of depression after 4 weeks (Table 6), and a long sleep in the other age groups can predict it after 3 weeks. The estimated coefficient of this week-lagged long sleep was 0.271 (Table 5). This implies that long sleep time increased K6 score from 5 to 6.86 after 3 weeks. Lagged long sleep also contributed to increase in the number of times lunch was not eaten in the present week. Table 6 shows that the estimated coefficient of 4-week lagged long sleep was 0.191, which was smaller than 0.271 above. Lagged long sleep in those aged 50–59 years had smaller effects on K6 scores this week, compared to the other age groups. Moreover, 4-week lagged UV light exposure had a decreasing effect on K6 this week.

## Discussion

The main findings of the present study were as follows. A PVAR analysis indicated that long sleep predicted the recurrence of depression after 3 weeks. Long sleep in patients aged 50–59 years predicted the recurrence of depression after 4 weeks, and long sleep in the other age groups predicted recurrence after 3 weeks.

Sleep disturbance is one of the diagnostic symptoms of major depression and is closely associated with depression, mainly in the form of insomnia (approximately 75%) and less often in the form of hypersomnia (approximately 10%) [24]. It is one of the most common residual symptoms of depression [25], which has been repeatedly found to constitute greater risk for subsequent depression recurrence [26, 27]. While it is usually residual insomnia that has been found to constitute a risk factor [28], some studies did find hypersomnia to be a risk factor as well [29]. In either case, however, they are sleep disturbance measured with an observer-rated or self-rated symptom inventory and therefore covering the past one or two weeks, our study was unique in measuring daily change in sleep hours and singling out long sleep, rather than short sleep, 3–4 weeks prior to depression aggravation.

Not eating lunch regularly, sitting idly and UV exposure (as a measure of outings) were candidate variables to predict depression relapse/recurrence. However, when taken together with long sleep, they were no longer predictive. The non-significant nature of their contributions may be due to low statistical power of the current sample (*n* = 89), and their joint predictive capabilities merit further investigation in a larger sample in the future. The current explanatory feasibility study has established that such a study is possible.

This study has important features. First, using the Kurashi-app, we were able to collect lifelog data from 89 patients for 1 year. While it is difficult for researchers to analyse sleep habits using conventional pen and paper methods, accurate information about sleep habits over longer sample periods allows for an easier empirical analysis. In general, sleep-diary data tend to be subjective daily reports of sleep from 1 to 2 weeks [30], which are data from shorter sample periods than the current data. Because the Kurashi-app extracts clusters of activities every 30 min, we were able to record a variety of activities such as sitting idly. From the viewpoint of the accuracy of activity records, our data of detailed activities, based on semi-automated recordings, corrected by the participants under close central monitoring, were superior to data of conventional studies that used several variants of Life Chart Method to examine mood course over longer periods of time (see, [31]).

Secondly, we used two screening tools for depression. By using both K6 and PHQ-9, we imputed the missing K6 data. Missing UV light exposure data were also imputed. We were able to define a pseudo-positive dummy variable as a false alarm of recurrence of depression, and used it to analyse the increase in K6 and PHQ-9 scores, although this increase was not necessarily indicative of a full depression recurrence above the diagnostic threshold.

Thirdly, we identified predictors of the deterioration of mental health status quantitatively through the estimation of PVAR models. Our panel VAR model was not liable to lead to seriously biased coefficients, compared to standard panel data methods such as fixed-effects model or random-effects model. This is a strength of our approach. Previous studies pointed out efficacy of early intervention aimed at preventing relapse or recurrence among high risk populations [32, 33]; however, no prior studies have established a method for identifying the signs of recurrence. If one could detect the signs of depression recurrence and perform a cognitive behavioural therapy intervention in a timely manner, the deterioration of mental health status could be minimised.

On the other hand, the study has some limitations. First, we did not restrict on medication and psychotherapy, and therapists could change their treatment depending on the situation. It might have influenced the relapse. However, our intention was to capture the approaching relapse even in such circumstances. Secondly, any prediction model require replication for extensive validation; however, we did not have a big enough sample size to ascertain external validity. We need future studies to examine replication of our findings. Third, the relapse/recurrence was defined by self-reports on K6. Finally, the convenient sampling of the present cohort may limit our ability to generalise findings from this study.

## Conclusion

We found that long sleep time was a risk factor for the recurrence of depression three to four weeks later. The PVAR approach using lifelog data could contribute to establishing a method for identifying the warning signs of recurrence in patients in remission of major depression. Future research could focus on identifying the predictors of long sleep time, which was one of the dependent variables in the PVAR model in the current study.

## Supplementary information

**Additional file 1.** Investigators and committee members.

## Data Availability

The datasets generated and/or analyzed during the current study are not publicly available due the Japanese clinical research ethics guidelines but are available from the corresponding author on reasonable request and after approval of the planned analyses by the ethics committee at Kyoto University Graduate School of Medicine as per the Japanese ethics guidelines.

## Abbreviations

K6: Kessler Psychological Distress Scale
PHQ-9: Patient Health Questionnaire-9
PVAR: Panel Vector Autoregressive
