# Supplementary material for: Predicting recurrence of depression using lifelog data: an explanatory feasibility study with a panel VAR approach
Source: BMC Psychiatry. 2019 Dec 11;19:391. doi: 10.1186/s12888-019-2382-2 (PMC6907185; doi:10.1186/s12888-019-2382-2)
Supplement: Supplementary file 1 — Additional file 1. Investigators and committee members. [file 12888_2019_2382_MOESM1_ESM.docx]

**Investigators and committee members**

**Steering Committee:** Toshi A. Furukawa (Chair and Principal Investigator, Kyoto University), Masaru Horikoshi (National Center of Neurology and Psychiatry), Tatsuo Akechi (Nagoya City University), Shinji Shimodera (Kochi University), Mitsuhiko Yamada (National Center of Neurology and Psychiatry), Masatoshi Inagaki (Okayama University), Norio Watanabe (Kyoto University), Hissei Imai (Kyoto University), Yusuke Ogawa (Kyoto University), Aran Tajika (Kyoto University)

**Site Principal and Co-principal Investigators:**

- Toho University: Masafumi Mizuno, Naohisa Tsujino
- Nagoya City University: Tatsuo Akechi, Megumi Uchida
- Hiroshima University: Yasumasa Okamoto, Ran Jinnin
- Kochi University: Shinji Shimodera, Hirokazu Fujita

**Participating Clinical Sites (number of participants recruited):**

- Toho Site (19): Bun Chino (Ginza Taimei Clinic), Naohisa Tsujino (Toho University)
- Nagoya Site (11): Megumi Uchida (Nagoya City University), Tadashi Funayama (Funayama Mental Clinic), Tatsuo Akechi (Nagoya City University), Yoshihiro Shinagawa (Shiki Clinic), Yoshio Ikeda (Narumi Himawari Clinic)
- Hiroshima Site (31): Ken’ichi Kurata (Kabe Mental Health Clinic ), Kyoka Ozaki (Morioka Clinic), Ran Jinnin (Hiroshima University), Yasumasa Okamoto (Hiroshima University)
- Kochi Site (39): Hirokazu Fujita (Kochi University), Hirotoshi Sato (Harimayabashi Clinic), Shinji Shimodera (Kochi University, Atago Hospital)
